# Psychometric properties of the Stress and Anxiety to Viral Epidemics-9 scale among frontline nursing professionals working in the COVID-19 inpatients ward

**DOI:** 10.3389/fpsyt.2022.934202

**Published:** 2022-07-22

**Authors:** Harin Kim, Jihoon Hong, Inn-Kyu Cho, Dongin Lee, Eulah Cho, Jin Yong Jun, Oli Ahmed, Seockhoon Chung

**Affiliations:** ^1^Department of Psychiatry, Asan Medical Center, University of Ulsan College of Medicine, Seoul, South Korea; ^2^University of Ulsan College of Medicine, Ulsan, South Korea; ^3^Department of Psychiatry, Ulsan University Hospital, University of Ulsan College of Medicine, Ulsan, South Korea; ^4^Department of Psychology, University of Chittagong, Chattogram, Bangladesh; ^5^National Centre for Epidemiology and Population Health, Australian National University, Canberra, ACT, Australia

**Keywords:** nurses, COVID-19, SAVE-9, anxiety, stress

## Abstract

This study examined the reliability and validity of the Stress and Anxiety to Viral Epidemics-9 (SAVE-9) scale among nursing professionals working in a COVID-19 inpatient ward. An anonymous, online survey was conducted among working frontline nursing professionals between April 7 and 18, 2022. We collected information about the participants' age, sex, years of employment, shift work, and marital status. In addition, the participants were asked whether they had dealt with infected patients recently, and whether they had been quarantined, infected, or vaccinated. SAVE-9, Generalized Anxiety Disorder-7 (GAD-7), and Patient Health Questionnaire-9 (PHQ-9) were used to evaluate symptoms. We used the Confirmatory Factor Analysis (CFA) to determine the validity of the two-factor model of the SAVE-9 scale. We also tested reliability and convergent validity using the PHQ-9 and GAD-7 scales. A total of 136 responses was analyzed, and CFA for two-factors model of the SAVE-9 scale showed a good model fit among frontline nursing professionals (CFI = 1.000, TLI = 1.040, RMSEA = 0.000, RSMR = 0.060). Multi-group CFAs revealed that the SAVE-9 scale can measure work-related stress and viral anxiety in the same way across sex, having depression, or having generalized anxiety. The internal consistency was shown to be good, and the SAVE-9 scale was significantly correlated with the GAD-7 (r = 0.328, *p* < 0.001) and PHQ-9 score (r = 0.361, *p* < 0.001). The two-factor model of the SAVE-9 is a valid and reliable scale for frontline nursing professionals.

## Introduction

Since the first outbreak of the coronavirus disease 2019 (COVID-19) in China, followed by the declaration of the pandemic on March 11, 2020, it has become one of the most significant global health problems. In South Korea, the first official case was reported on January 20, 2020 and the cumulative number of COVID-19 patients increased steadily, reaching up to 16,471,940 cases on April 19, 2022 (1). Psychosocial risk factors for social isolation inevitably increased because of the pandemic, which, in turn, led to a 25% increase of global prevalence of depression and anxiety. In addition, exhaustion became a major triggering factor of suicidal ideation among healthcare providers (2).

Healthcare professionals have been at a greater risk of work overload amidst the pandemic (3), and a shortage of nursing staff is indicated when compared to other medical situations in Korea (4). Therefore, many studies have focused on the occupational stress of healthcare professionals involved in the COVID-19 (5, 6). The stress might be closely related to psychological burden. As they are easily exposed to the risk of viral infection, they are more likely to pass it to their family members or even other immunocompromised patients at the hospital. Moreover, the use of protective equipment imposes significant physical exhaustion on medical staff. Our experience substantially demonstrates that COVID-19 frontline nurses frequently report physical and mental fatigue because of limited chances of drinking water or using the toilet.

In particular, frontline nurses who were caring for COVID-19 patients reported that they experienced significant anxiety, which was related to their stress from work, whereas their rights were relatively ignored in the crisis (7, 8). Similarly, research on psychiatric problems of healthcare workers treating other infectious diseases showed that they were vulnerable to severe emotional distress, depression, anxiety, and even posttraumatic stress disorder (9, 10). During the influenza pandemic, most healthcare professionals said that they were willing to take care of patients, even as they were generally expected to take the risk of being infected with the virus (11). Moreover, psychological burden was most profound among nurses when compared to other medical staff during the outbreak of Severe Acute Respiratory Syndrome (12). Therefore, nurses would often face the ethical dilemma of taking the risks as expected, or changing their duties or even quitting from the hospital.

The emotional distress is associated with poor job performance of nurses. Their workload and frustration had a negative impact on their performance during the COVID-19 pandemic (13). In addition, long-term exposure to the fear of viral infection led to nurses' exhaustion, which, in turn, led to a decision to leave their job during the outbreak of infectious disease (14). Hence, an evaluation of stress and anxiety of nurses who work in infectious disease wards is essential in terms of patient's safety and the management of human resources.

Recently, we developed the Stress and Anxiety to Viral Epidemics-9 items (SAVE-9) scale to assess healthcare workers' work-related stress and anxiety in response to viral epidemics (15). The SAVE-9 scale (www.save-viralepidemic.net) was validated in Korean (15), Italian (16), Russian (17), German (18), Japanese (19), and Turkish (20). Studies had been conducted among healthcare workers including medical doctors, nursing professionals, and other healthcare workers in general hospitals, but none of them focused on frontline nursing professionals working in the COVID-19 inpatients wards. Though the SAVE-9 scale was validated among various healthcare workers in numerous countries, the reliability and validity need to be explored again among frontline nursing professionals working in COVID-19 inpatients wards, who suffer from severe long-lasting stress while taking care of infected people with personal protective equipment. South Korea, especially, reports over 100,000 infections per day and has experienced a rapid increase in the number of inpatients with COVID-19. The stress experienced by frontline nursing professionals in COVID-19 wards should be explored and managed. This study explored the reliability and validity of the SAVE-9 scale among front-line nursing professionals working in the COVID-19 inpatients ward.

## Materials and methods

### Participants and procedure

This anonymous online survey study was conducted among frontline nursing professionals working in the COVID-19 inpatients wards at the Asan Medical Center during April 7–18, 2022. In February 2022, the Center for Infection Control (CIC) opened in Asan Medical Center, and it is the first time that a Korean private hospital had a separate facility wholly dedicated to infectious diseases (21). Nursing professionals voluntarily participated in the survey, and we provided a 10 US dollar-valued gift coupon for the participation. No personal information was gathered in this study. The protocol for this survey study was approved by the Institutional Review Board (2022–0323) of the Asan Medical Center, and obtaining the written informed consent for participation was waived.

In this survey, participants' information on age, sex, years of employment, shift-working and marital status were collected. The participants also were requested to respond to questions related to COVID-19, such as whether they had been taking care of infected patients and were quarantined, infected, or getting vaccinated. Responses to questions on their past psychiatric history and current psychiatric distress were collected. We developed the e-survey form according to the Checklist for Reporting Results of Internet e-Surveys (CHERRIES) guidelines (22), and the usability and technical functionality were tested prior to the survey.

Sample size estimation was done based on the rule of 10:1 (ideal ratio of respondents to items) (23). As the SAVE-9 scale includes 9 items, a sample size of at least 90 was needed. The hospital has 239 nursing professionals working in the 6 wards for COVID-19 patients. We aimed to enroll 143 (60.0%) participants, and 139 (58.2%) nursing professionals responded to the online survey.

### Symptom assessment

#### Stress and anxiety to viral epidemics-9 items

The SAVE-9 scale was developed to measure healthcare workers' work-related stress and anxiety in response to the COVID-19 pandemic (15). Originally, nine items of the SAVE-9 were clustered into two factors: factor I—anxiety about the epidemic (SAVE-6; items 1, 2, 3, 4, 5, and 8) (24); and factor II—work-related stress associated with the epidemic (SAVE-3; items 6, 7, and 9) (25). The items of SAVE-9 can be rated on a 5-point Likert scale; 0 (never) - 4 (always), and a higher score reflects the higher level of stress and anxiety due to the viral epidemic. In the previous study (25), reliability of internal consistency was good (Cronbach's alpha = 0.80 and McDonald's omega = 0.81). The cut-off points of SAVE-9 and factor I (SAVE-6) scale were examined as 22 and 15 according to the mild degree of Generalized Anxiety Disorder-7 items (GAD-7) scale (GAD-7 ≥ 5) (15). The SAVE-9 scale was originally developed in the Korean language, and we applied the Korean version in this study.

#### Generalized anxiety disorder-7 items

GAD-7 is a self-rating questionnaire designed to measure an individual's level of general anxiety. the seven items of the GAD-7 scale can be rated on a 4-point Likert scale which range from 0 to 3 (0 = not at all to 3 = nearly every day), and a higher score means a severe degree of anxiety symptoms (26). This study applied the Korean version of the GAD-7 (27), and we observed the Cronbach's alpha value to be 0.908 in this sample.

#### Patient health questionnaire-9 items

The PHQ-9 is a self-rating questionnaire designed to measure an individual's level of depressive symptoms. The nine items of the PHQ-9 can be scored on a 4-point Likert scale which range from 0 to 3 (0 = not at all to 3 = nearly every day), and a higher score means a more severe degree of depressive symptoms (28). This study applied the Korean version of the PHQ-9 (27), and we observed a Cronbach's alpha value of 0.851 in this sample.

### Statistical analysis

We conducted a Confirmatory Factor Analysis (CFA) (estimation method: DWLS) to assess the validity of the two-factor model used in the previous study (29). The two factor model of the SAVE-9 scale was already explored in the previous studies (15, 25). In this study, we did not conduct the exploratory factor analysis, since we tried to confirm whether the SAVE-9 can measure stress and anxiety of nursing professionals who are working in COVID-19 inpatients ward with a good model fits for two-factor model. Prior to CFA, the normality assumption of all 9 items were checked based on skewness and kurtosis for an acceptable limit of range ± 2 (30). We ran multigroup CFA to assess the measurement invariance across sex, having depression [PHQ-9 ≥ 10 (28)] and anxiety [GAD-7 ≥ 10 (26)]. We also assess the psychometric properties of this scale using the Item Response Theory (IRT) Approach through Graded Response Model (GRM) and Rasch model. Before running GRM, we assessed IRT assumptions—local dependence [*p*-values (adjusted for false discovery rate) of G^2^] and monotonicity (the number of significant violations and *Crit* value). Unidimensionality was not assessed as the scale is not unidimensional. In GRM, we first assessed item fits through S-χ^2^ and its *p*-values (adjusted for false discovery rate). Subsequently, the slope parameters (α) and threshold parameters (b) of the items were assessed and the scale information curve of the SAVE-9 scale and item characteristic curves were extracted. IRT reliability was also calculated. In Rasch Model, infit mean square (infit MnSQ), outfit MnSQ, item difficulty, item and person separation index, and item and person reliability were estimated. Reliability test of the SAVE-9 scale was done using Cronbach's alpha and McDonald's Omega. To explore the convergent validity, we performed Pearson's correlation analysis with PHQ-9 and GAD-7 scales. The SPSS version 21.0, RStudio, and jMetrik softwares were utilized for statistical analyses.

## Results

Of the 139 study participants, 136 respondents, who comprised 91.9% female and 8.1% male nursing professionals, completed the survey and thus were included in further analysis (Table 1). Their mean age was 30.2 ± 5.6 years old and mean duration of employment was 7.2 ± 5.6 years. Approximately 61.7% of participants were single and 97.8% were working as shift workers in the hospital. As for psychiatric history, 16.2% had a history of depression, anxiety or insomnia whereas 9.6% reported being depressed or anxious at the time. Concerning questions related to the COVID-19, all of them were fully vaccinated and taking care of inpatients with COVID-19; 40.4% had been quarantined and 35.3% had been diagnosed with COVID-19. Notably, 83.1% reported that they experienced patients' deaths because of the disease. Among the participants, 41 (30.1%) were rated as having depression (PHQ-9 ≥ 10), and 55 (40.4%) nursing professionals were found to suffer high levels of stress and anxiety, which correspond to 22 points on the SAVE-9 scale (15).

**Table 1:** Clinical characteristics of participants (*N* = 136).

**Variables**	***N*** **(%) Mean** ±**SD**
Sex (female)	125 (91.9%)
Age	30.2 ± 5.6
Years of employment	7.2 ± 5.6
Marital status*	
Single	103 (61.7%)
Married, without kids	14 (8.4%)
Married, with kids	18 (10.8%)
Are you a shift worker? (Yes)	133 (97.8%)
Questions on COVID-19	
Are you taking care of COVID-19 infected patients? (Yes)	136 (100.0%)
Did you experience being quarantined due to infection with COVID-19? (Yes)	55 (40.4%)
Did you experience being infected with COVID-19? (Yes)	48 (35.3%)
Did you get vaccinated? (Yes)	136 (100.0%)
Did you experience deaths of COVID-19 infected patients? (Yes)	113 (83.1%)
Psychiatric history	
Did you have experience or treated depression, anxiety, or insomnia? (Yes)	22 (16.2%)
Now, do you think you are depressed or anxious, or do you need help for your mood state? (Yes)	13 (9.6%)
Rating scales scores	
Stress and Anxiety to Viral Epidemic-9 items	20.6 ± 6.4
Factor I (SAVE-6)	14.2 ± 4.9
Factor II (SAVE-3)	6.3 ± 2.6
Generalized Anxiety Disorders-7 items	7.3 ± 4.8
Patient Health Questionnaire-9 items	3.2 ± 3.8

*There was one missing value.

### Factor structure of SAVE-9 among nursing professional working in COVID-19 inpatients ward

**Figure 1:**
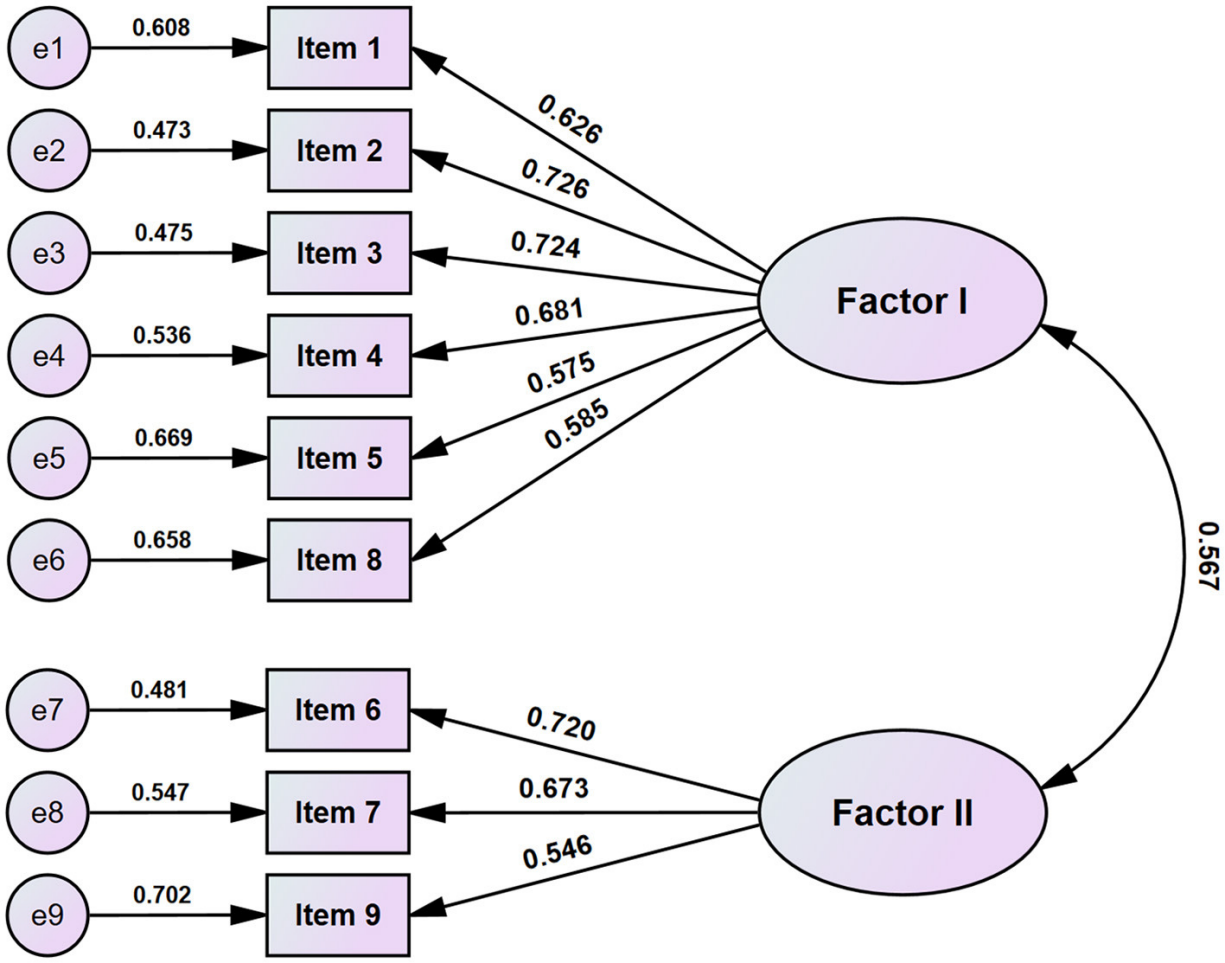
Factor structure of the SAVE-9 scale among nursing professionals working in COVID-19 inpatients ward.

Before CFA, we ensured that all 9 items of the SAVE-9 scale were distributed within the normal limit based on the skewness and kurtosis (Table 2). We conducted CFA for two-factors model of the SAVE-9 scale (Figure 1) according to the previous study (15), and observed a good model fit among nursing professionals working in COVID-19 inpatients wards (CFI = 1.000, TLI = 1.040, RMSEA = 0.001, RSMR = 0.060). The multi-group CFAs with configural invariance showed that the SAVE-9 scale can measure work-related stress and viral anxiety in the same way across sex (male vs. female, CFI = 1.000, RMSEA = 0.001), having depression (PHQ-9 ≥ 10, CFI = 1.000, RMSEA = 0.001) or having generalized anxiety (GAD-7 ≥ 10, CFI = 1.000, RMSEA = 0.001) (Supplementary Table 1). Similar results were shown in multi-group CFAs with metric or scale invariant models.

**Table 2:** Item properties of the SAVE-9 scale among nursing professionals working in COVID-19 inpatients wards.

**Items**	**Response scale**					**Descriptive**				**CITC**	**CID**	**Factor loadings (CFA)**	
	**0**	**1**	**2**	**3**	**4**	**M**	**SD**	**Skewness**	**kurtosis**			**Factor I** **(SAVE-6)**	**Factor II (SAVE-3)**
Item 1	6.6	8.8	16.9	53.7	14.0	2.60	1.05	−0.998	0.536	0.548	0.792	0.626	–
Item 2	8.1	11.8	22.8	40.4	16.9	2.46	1.15	−0.625	−0.333	0.671	0.764	0.726	–
Item 3	5.1	9.6	22.1	41.9	21.3	2.65	1.08	−0.729	0.037	0.650	0.770	0.724	–
Item 4	5.9	18.4	18.4	39.7	17.6	2.45	1.15	−0.475	−0.694	0.624	0.775	0.681	–
Item 5	31.6	40.4	7.4	13.2	7.4	1.24	1.24	0.904	−0.249	0.465	0.813	0.575	–
Item 6	13.2	26.5	25.0	23.5	11.8	1.94	1.23	0.065	−0.984	0.572	0.453	–	0.720
Item 7	14.0	36.0	29.4	19.1	1.5	1.58	1.00	0.157	−0.753	0.537	0.520	–	0.673
Item 8	5.9	7.4	11.8	46.3	28.7	2.85	1.10	−1.107	0.711	0.522	0.797	0.585	–
Item 9	2.9	11.0	17.6	41.2	27.2	2.79	1.06	−0.746	−0.067	0.369	0.717	–	0.546

*0, Never; 1, Rarely; 2, Sometimes; 4, Often; 5, Always; M, Mean; SD, Standard Deviation; CITC, Corrected Item-Total Correlation; CID, Cronbach's Alpha if Item Deleted*.

### Graded response model

Supplementary Table 2 presents the results about IRT assumptions. The *p*-values (adjusted for false discovery rate) of G^2^ are non-significant at 0.01. There is also an absence of significant violation of monotonicity and crit values are zero. Therefore, IRT assumptions are met. Supplementary Table 3 presents the GRM outputs. Non-significant (≥0.05) *p*-values (adjusted for false discovery rate) of the S-χ^2^ suggest that all the items belong to the same latent construct. In Factor I, item 5 has moderate slope, item 1 and item 8 have high slope and the rest of the three items have a very slope. Slope parameters (α) range between 1.207 and 2.871 (mean α = 1.836) for Factor I. About threshold coefficients (b) in Factor I, item 5 is the most difficult item compared to other items. A higher latent trait or theta is required to endorse response options “always” in all items except item 5. Item characteristic curves graphically depicted this information. In Factor II, Item 9 has moderate slope and the rest of the two items have very slopes. Slope parameters (α) range between 0.869 and 2.521 (mean α = 1.892) for Factor II. About threshold coefficients (b) in Factor II, item 9 is the least difficult item and item 7 is the most difficult item. Item characteristic curves (Supplementary Figures 1, 2) graphically depicted this information. Scale information curve (Supplementary Figure 3) factors show that Factor I provides more information than Factor II.

### Rasch outputs

Supplementary Table 4 presents the Rasch model outputs of the SAVE−9 scale for the health care workers. Infit and outfit mean squares of all the items are within the recommended range (0.50–1.50). About item difficulty in Factor I, item 8 is the least difficult item and item 5 is the most difficult item. Item and person reliability and separation indices are also above the recommended cut off (>2 and ≥0.7, respectively). About item difficulty in Factor II, item 9 is the least difficult item and item 7 is the most difficult item. Item and person reliability of Factor II is above the recommended cut off. Although the item separation index for Factor II is above the recommended cut off, person separation index is below the recommended cut off.

### Reliability and evidence based on relations to other variables of the SAVE-9 scale among nursing professionals working in COVID-19 inpatients wards

The internal consistency of the SAVE-9 scale (Cronbach's alpha = 0.818, McDonald's Omega = 0.815), factor I (Cronbach's alpha = 0.815, McDonald's Omega = 0.816), or factor II (Cronbach's alpha = 0.672, McDonald's Omega = 0.710) was shown to be good (Table 3). Item-total correlation for factor I ranged between 0.465 and 0.671, and that for factor II ranged between 0.369 and 0.572. Each factor of this scale also had good IRT reliability (0.843 and 0.756). The SAVE-9 scale was significantly correlated with the GAD-7 (r = 0.328, *p* < 0.001) and PHQ-9 (r = 0.361, *p* < 0.001) scores. Factor I and factor II scores were also significantly correlated with GAD-7 (r = 0.280, r = 0.283, all *p* < 0.001) and PHQ-9 (r = 0.287, r = 0.366, all *p* < 0.001).

**Table 3:** Scale level psychometric properties of the SAVE-9 scale among nursing professionals working in COVID-19 inpatients wards.

**Psychometric properties**	**SAVE-9**			**Suggested cut off**
	**Factor 1**	**Factor 2**	**Overall**	
Floor effect			0.7	15%
Ceiling effect			0	15%
Mean inter-item correlation	0.426	0.406	0.334	Between 0.15 and 0.50
Cronbach's alpha	0.815	0.672	0.818	≥0.7
McDonald's Omega	0.816	0.710	0.815	≥0.7
Split-half reliability (odd-even)	0.848	0.559	0.863	≥0.7
Standard error of measurement			2.74	< SD, (6.43)/2
*Rho* coefficient	0.830	0.694		≥0.7
IRT reliability	0.843	0.756		≥0.7
Item separation index	5.94	5.66		≥2
Person separation index	2.06	1.63		≥2
Item reliability	0.973	0.970		≥0.7
Person reliability	0.810	0.726		≥0.7
Model fits of confirmatory factor analysis				
*χ^2^* (df, *p*-value)	39.117 (52, 0.906)			Non-significant
CFI	1.000			>0.95
TLI	1.040			>0.95
RMSEA	0.001			<0.08
SRMR	0.062			<0.08

## Discussion

In this study, we observed that 30.1 and 40.4% of nursing professionals working in COVID-19 inpatient wards in a tertiary-level general hospital in Korea were rated as having depression or high levels of work-related stress and anxiety response to viral epidemics in this COVID-19 era, respectively. The two-factor model of the SAVE-9 showed a good model fit among frontline nursing professionals, and the internal consistency of the SAVE-9 scale and its two factors (factor I—SAVE-6 and factor II—SAVE-3) was shown to be good. In GRM, one item in each factor had a moderate slope coefficient. However, items provided reasonable information about discrimination between low scorers and high scorers in the SAVE-9 for the health care workers. The Rasch model provided similar information as the GRM.

Originally, the SAVE-9 scale was developed for medical doctors, nurses, and other healthcare professionals working in a general hospital (15). Our goal was to create a rating scale that could measure healthcare workers' work-related stress and anxiety responses specifically to viral epidemics, and we wanted to create a rating scale that could be applied to all healthcare workers, regardless of their duties, role, or jobs. Not all healthcare workers who had participated in the validation studies were experienced in taking care of infected patients. The participants in an Italian study reported being responsible for cases of COVID-19 infection (16), and in a German study, 75.4% reported that they directly experienced COVID-19 patients (18). However, no specific information on taking care of infected cases was presented in Russian (17), Japanese (19), Turkish (20) and Korean (15) studies. Furthermore, work-related stress or anxiety level was repeatedly reported to be high among nursing professionals compared to other healthcare workers. Nursing is a female-dominated profession, and depression and anxiety are more prevalent in females (31), Nurses often work three shifts each week, which can lead to insomnia and anxiety (32). Moreover, a high staff turnover rate can make it difficult to adapt to new protocols. It was repeatedly reported that nurses experienced psychological distress during the COVID-19 pandemic (33). Therefore, the development of a stress management system is essential for healthcare workers, especially during the prevailing pandemic. In addition, this study tested the reliability and validity of the SAVE-9 scale specifically among nursing professionals working in COVID-19 inpatients wards.

Originally, the SAVE-9 scale was clustered into two factors. Factor I, which consists of items 1, 2, 3, 4, 5, and 8, was related to viral anxiety; we applied it to measure an individual's viral anxiety among the general population (namely, SAVE-6 scale) (24). Factor II, consisting of items 6, 7, and 9, was related to work-related stress in response to the viral epidemic; we applied it to assess healthcare workers' work stress during the COVID-19 pandemic (25). In this study, we also observed that a two-factor model of the SAVE-9 scale can be reliably applied to assess nursing professionals working in COVID-19 inpatients wards. Previous studies showed similar results of two factor models of the SAVE-9 scale (16, 19, 20), but those conducted in Russia and Germany showed different factor models (17, 18). In a Russian study, factor I (anxiety about the viral epidemic) included items 2, 3, 4, and 8; and factor II (work-related stress associated with the viral epidemic) included items 1, 5, 6, 7, and 9. In a German study, the factor model found in Russia showed better fit indices compared to the original factor model, though the original factor model also showed a good fit.

The discrepancy between the factor model may come from cultural differences. The cultural differences between European (Russia and Germany) and Asian countries (Korea, Japan, or Turkey) might influence the results. The clustering of item 5 (Are you worried that others might avoid you even after the infection risk has been minimized?) is a major issue. It was inspired by a concern that healthcare workers have about being stigmatized by their community. In usual disasters, healthcare workers provide assistance to patients injured in the disaster but are not directly impacted. They can, however, be directly affected during an epidemic, and be infected from the patients. Healthcare workers who come into close contact with patients with COVID-19 are at risk of acquiring the disease if they lack proper protective equipment. They are overloaded, have poor infection control, and have pre-existing medical conditions (34). Their neighbors are concerned about viral transmission from them, which causes healthcare workers to feel anxious or stressed. Across cultures, the attitude toward item 5 seems to differ. Among Spanish healthcare workers, the factor loading value of item 5 was very low (0.38) when the SAVE-6 scale was applied (35). Even in a Peruvian study, SAVE-6 was applied to medical students after excluding item 5, as the factor loading value of item 5 was too low (0.251) (36).

This study explored the scale information curve of SAVE-9 and its two subscales (SAVE-6 and SAVE-3). We observed that both SAVE-9 and SAVE-6 scales provide almost the same level of information. However, the SAVE-3 scale produced relatively lesser information. If we take the results into consideration, the application of SAVE-6 is comparable to that of SAVE-9 to measure stress and anxiety in healthcare workers. In a previous study, we assessed the SAVE-6 scale among healthcare workers in Spain (35), and concluded that the SAVE-6 scale is a reliable and valid way to evaluate healthcare workers' anxiety during the COVID-19 pandemic. A full validation of SAVE-3 has not been conducted among various groups of samples. We examined the reliability and validity of the SAVE-3 scale in relation to the Maslach Burnout Inventory in one study. Further study is needed to validate the scale in other groups.

This study was limited by several factors. It was conducted among nursing professionals in a single private hospital, so the results cannot be generalized. Nevertheless, South Korea's ASAN Medical Center is the nation's largest single tertiary-level hospital, which has a separate building dedicated exclusively to infectious disease. It is advantageous to gather the unique homogenous group in this hospital. Second, an anonymous online survey might lead to bias. Nonetheless, online surveys need to be conducted instead of face-to-face interviews to avoid viral transmission in this pandemic era. Third, this study was conducted 2 years after the onset of the COVID-19 pandemic. The participants may have adjusted to the situation, which might have affected the results. However, over 100,000 infected cases were reported per day, and the number of inpatients surged, which meant that the workload continued to increase. Fourth, most of participants (91.9%) were women in this study. Female preponderance of this sample might leads to bias, since high level of viral anxiety was observed among female participants in the previous studies (37, 38). Finally, the small sample size of 136 nursing professionals may limit the generalizability of the results. However, we tried to collect at least 60% (*N* = 143) of All 238 nursing professionals were working in COVID-19 inpatients wards of Asan Medical Center.

In conclusion, we found that the SAVE-9 scale and its subscales SAVE-6 and SAVE-3 can be used to assess work-related stress and viral anxiety among frontline nurses working in COVID-19 inpatient units. Assessing and managing their stress or viral anxiety will allow them to adjust to the stressful situation and ensure their safety and that of the patients they care for.

## Data availability statement

The raw data supporting the conclusions of this article will be made available by the authors, without undue reservation.

## Ethics statement

The protocol for this survey study was approved by the Institutional Review Board (2022-0323) of the Asan Medical Center, and obtaining the written informed consent for participation was waived. Written informed consent for participation was not required for this study in accordance with the national legislation and the institutional requirements.

## Author contributions

SC, HK, and JJ: conceptualization, investigations, and resources. SC, HK, JJ, DL, I-KC, and EC: data curation. SC, OA, and HK: formal analysis. SC and OA: methodology. SC, JJ, and HK: project administration. I-KC: visualization. HK, DL, I-KC, EC, SC, JJ, and OA: writing—original draft. All authors: writing—review and editing. All authors contributed to the article and approved the submitted version.

## Funding

This work was supported by the Student Research Grant (2022-0629) of University of Ulsan, College of Medicine, Seoul, Korea.

## Conflict of interest

The authors declare that the research was conducted in the absence of any commercial or financial relationships that could be construed as a potential conflict of interest.

## Publisher's note

All claims expressed in this article are solely those of the authors and do not necessarily represent those of their affiliated organizations, or those of the publisher, the editors and the reviewers. Any product that may be evaluated in this article, or claim that may be made by its manufacturer, is not guaranteed or endorsed by the publisher.
