# Supplementary material for: Psychometric properties of the Stress and Anxiety to Viral Epidemics-9 scale among frontline nursing professionals working in the COVID-19 inpatients ward
Source: Front Psychiatry. 2022 Jul 22;13:934202. doi: 10.3389/fpsyt.2022.934202 (PMC9353028; doi:10.3389/fpsyt.2022.934202)
Supplement: Supplementary file 1 [file Data_Sheet_1.docx]

**Supplementary Table 1. Measurement invariance**

| **Model** | **χ^2^** | **df** | **Δ χ^2^** | **Δdf** | **p** | **CFI** | **ΔCFI** | **RMSEA** | **ΔRMSEA** |
| --- | --- | --- | --- | --- | --- | --- | --- | --- | --- |
| **Sex (male vs. female)** | | | | | | | | | |
| **Configural** | 28.255 | 52 |  |  |  | 1.000 |  | .001 |  |
| **Metric** | 35.587 | 59 | 7.332 | 7 | 0.395 | 1.000 | 0 | .001 | 0 |
| **Scalar** | 39.465 | 66 | 3.878 | 7 | 0.794 | 1.000 | 0 | .001 | 0 |
| **Depression (PHQ-9 ≥ 10 vs. PHQ-9 < 10)** | | | | | | | | | |
| **Configural** | 38.731 | 52 |  |  |  | 1.000 |  | .001 |  |
| **Metric** | 55.092 | 59 | 16.361 | 7 | 0.022 | 1.000 | 0 | .001 | 0 |
| **Scalar** | 60.350 | 66 | 5.258 | 7 | 0.629 | 1.000 | 0 | .001 | 0 |
| **Generalized anxiety (GAD-7 ≥ 10 vs. GAD-7 < 10)** | | | | | | | | | |
| **Configural** | 39.117 | 52 |  |  |  | 1.000 |  | .001 |  |
| **Metric** | 46.324 | 59 | 7.207 | 7 | 0.408 | 1.000 | 0 | .001 | 0 |
| **Scalar** | 54.053 | 66 | 7.729 | 7 | 0.357 | 1.000 | 0 | .001 | 0 |

**Supplementary Table 2. Monotonicity and G^2^ p values of items of the SAVE-9 scale**

| Items | **Monotonicity** | | | | **Local dependance G^2^ p values** | | | | | | |
| --- | --- | --- | --- | --- | --- | --- | --- | --- | --- | --- | --- |
|  | **#ac** | **#vi** | **#zsig** | ***Crit*** | **Item 1** | **Item 2** | **Item 3** | **Item 4** | **Item 5** | **Item 6** | **Item 7** |
| **Item 1** | 4 | 0 | 0 | 0 |  |  |  |  |  |  |  |
| **Item 2** | 3 | 0 | 0 | 0 | .488 |  |  |  |  |  |  |
| **Item 3** | 4 | 0 | 0 | 0 | .601 | .726 |  |  |  |  |  |
| **Item 4** | 4 | 0 | 0 | 0 | .014 | .726 | .571 |  |  |  |  |
| **Item 5** | 3 | 0 | 0 | 0 | .033 | .122 | .060 | .778 |  |  |  |
| **Item 8** | 4 | 0 | 0 | 0 | .033 | .060 | .584 | .605 | .601 |  |  |
| **Item 6** | 4 | 0 | 0 | 0 |  |  |  |  |  |  |  |
| **Item 7** | 3 | 0 | 0 | 0 |  |  |  |  |  | .404 |  |
| **Item 9** | 4 | 0 | 0 | 0 |  |  |  |  |  | .404 | .404 |
| ac = active comparison, vi = violation, zsig = significant violation  Notes: p-values adjusted for false discovery rate (FDR) | | | | | | | | | | | |

**Supplementary Table 3. Item fit, and slope and threshold parameters of the SAVE-9 scale**

| **Items** | | **Item fits** | | | **Slope parameter (a)** | **Threshold parameter (b)** | | | |
| --- | --- | --- | --- | --- | --- | --- | --- | --- | --- |
|  |  | **S-χ^2^** | **df** | **p value** |  | **b_1_** | **b_2_** | **b_3_** | **b_4_** |
| **Factor I**  **(SAVE-6)** | **Item 1** | 25.873 | 17 | .192 | 1.641 | -2.242 | -1.385 | -.571 | 1.554 |
|  | **Item 2** | 8.623 | 13 | .818 | 2.871 | -1.644 | -.902 | -.163 | 1.077 |
|  | **Item 3** | 22.472 | 15 | .192 | 2.198 | -2.140 | -1.294 | -.389 | .979 |
|  | **Item 4** | 18.230 | 18 | .662 | 1.755 | -2.288 | -.923 | -.231 | 1.269 |
|  | **Item 5** | 33.381 | 20 | .186 | 1.207 | -.810 | .902 | 1.305 | 2.491 |
|  | **Item 8** | 14.233 | 20 | .818 | 1.342 | -2.605 | -1.751 | -1.029 | .895 |
| **Factor II**  **(SAVE-3)** | **Item 6** | 7.718 | 5 | .172 | 2.521 | -1.376 | -.267 | .511 | 1.427 |
|  | **Item 7** | 4.514 | 5 | .478 | 2.287 | -1.368 | .046 | 1.020 | 2.719 |
|  | **Item 9** | 13.732 | 8 | .089 | .869 | -4.406 | -2.337 | -1.009 | 1.311 |
| Notes: p-values adjusted for false discovery rate (FDR) | | | | | | | | | |

**Supplementary Table 4. Item fits and difficulties of the SAVE-9 scale through Rasch model**

| **Items** | | **Infit MnSq** | **Outfit MnSq** | **Difficulty** |
| --- | --- | --- | --- | --- |
| **Factor I**  **(SAVE-6)** | **Item1** | .95 | 1.05 | -.27 |
|  | **Item2** | .78 | .81 | -.09 |
|  | **Item3** | .83 | .84 | -.35 |
|  | **Item4** | .89 | .95 | -.07 |
|  | **Item5** | 1.13 | 1.09 | 1.43 |
|  | **Item8** | 1.23 | 1.21 | -.66 |
| **Factor II**  **(SAVE-3)** | **Item 6** | .90 | .88 | .24 |
|  | **Item 7** | .86 | .85 | .74 |
|  | **Item 9** | 1.35 | 1.29 | -.97 |

**
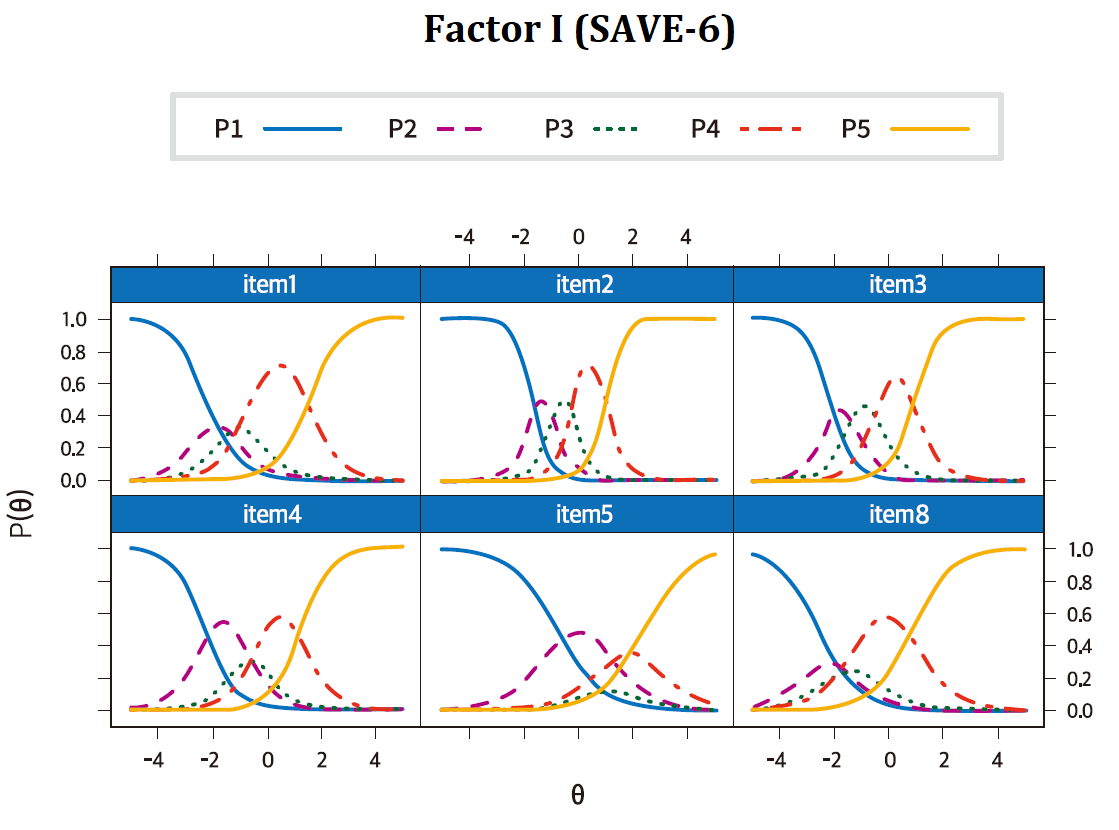
**

**Supplementary Figure 1. Item’s threshold curves of the factor I (SAVE-6)**

**
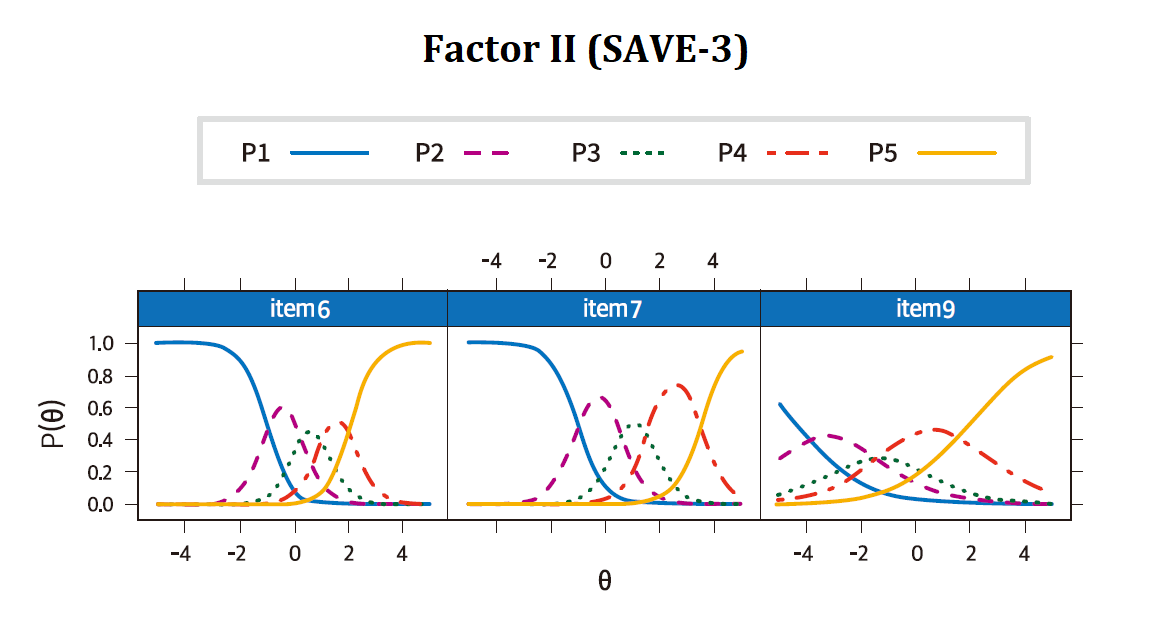
**

**Supplementary Figure 2. Item’s threshold curves of the factor II (SAVE-3)**

**
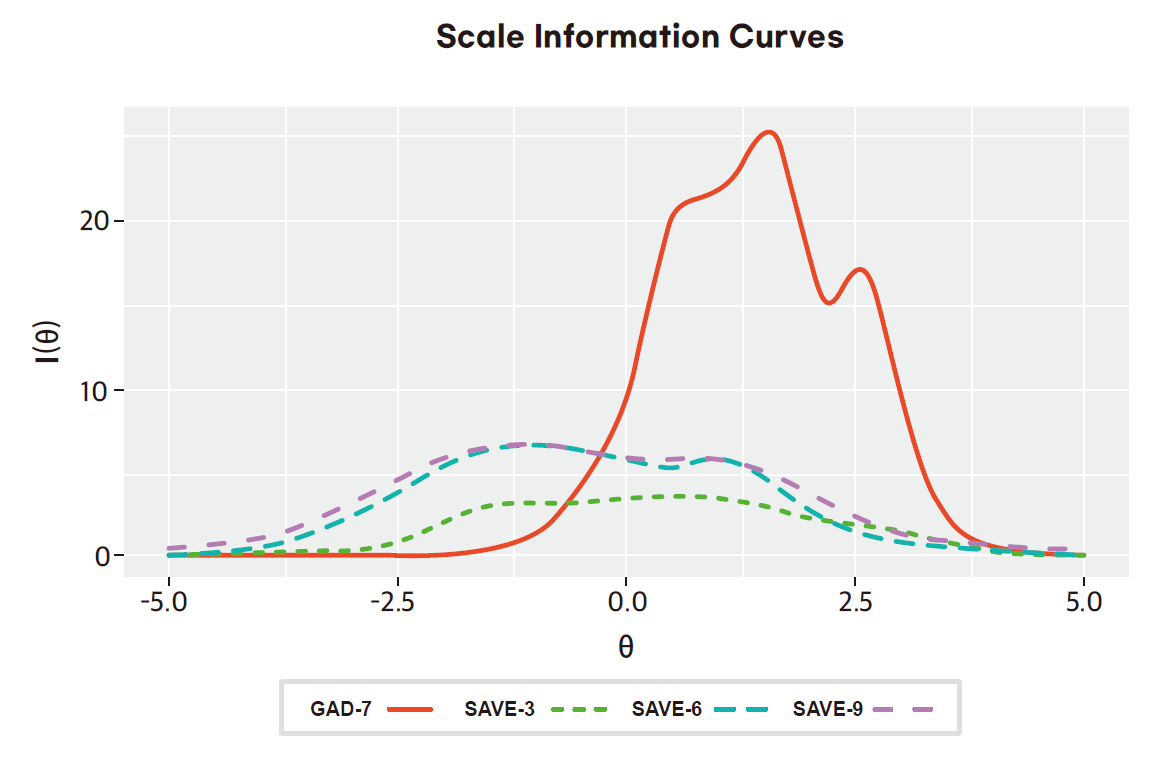
**

**Supplementary Figure 3. Scale information curve**
